# Comparative Effectiveness of Pomalidomide-Based Regimens in Relapsed/Refractory Multiple Myeloma: A Multicenter Real-World Analysis in China

**DOI:** 10.3390/cancers18071160

**Published:** 2026-04-03

**Authors:** Shan Gao, Junling Zhuang, Aijun Liu, Dongmei Wang, Wei Wang, Xin Li, Zhihong Wang, Meiyun Fang, Ming Gong, Zhilin Jia, Sun Wu, Zheng Xu, Genjie Wang, Li Bao

**Affiliations:** 1 Hematology Department, Beijing Jishuitan Hospital, Capital Medical University, Xicheng District, Beijing 100035, China; 2 Hematology Department, Peking Union Medical College Hospital, Beijing 100023, China; 3 Hematology Department, Beijing Chaoyang Hospital, Capital Medical University, Beijing 100020, China; 4 Hematology Department, Hengshui People’s Hospital, Hengshui 053000, China; 5 Hematology Department, The Affiliated Hospital of Qingdao University, Qingdao 266005, China; 6 Hematology Department, Fuzhou University Affiliated Provincial Hospital, Fuzhou 350001, China; 7 Hematology Department, Zhongshan Hospital Affiliated to Dalian University, Dalian 116001, China; 8 Hematology Department, China-Japan Friendship Hospital, Beijing 100029, China; 9 Hematology Department, The First Affiliated Hospital of Dalian Medical University, Dalian 116011, China; 10 Hematology Department, The First Affiliated Hospital of Xinxiang Medical University, Xinxiang 453199, China; 11 Hematology Department, Xinyang Central Hospital, Xinyang 464099, China; 12 Hematology Department, The First People’s Hospital of Shangqiu City, Shangqiu 476100, China

**Keywords:** multiple myeloma, pomalidomide, relapse, refractory, multicenter analysis

## Abstract

**Background**: Although multiple pomalidomide-based combinations are active in relapsed and/or refractory multiple myeloma (RRMM), comparative data to guide regimen selection remain limited. **Methods**: A total of 230 patients with RRMM from 12 centers in China who received pomalidomide-based regimens were included in this retrospective analysis. Overall response rate (ORR) and progression-free survival (PFS) were compared across regimens incorporating bortezomib or ixazomib (V/IPD), carfilzomib (KPD), or daratumumab (DPD), and multivariable analyses were performed to identify prognostic factors. **Results**: The overall ORR was 73.9%, with rates of 63%, 79%, and 85% in the V/IPD (*n* = 66), KPD (*n* = 69), and DPD (*n* = 95) cohorts, respectively. ORR differed significantly between V/IPD and DPD (*p* = 0.0165), driven by a higher proportion of ≥VGPR in the DPD group. The median PFS for the entire cohort was 17.4 months (95% CI: 13.7–20.1), compared with 15.4 months (95% CI: 12.8–20.5), 14.2 months (95% CI: 6.9–not estimable), and 19.2 months (95% CI: 15.1–24.9) for V/IPD, KPD, and DPD, respectively, without significant differences. In multivariable analysis, DPD was associated with improved ORR (HR 4.83, *p* < 0.001) but not with PFS. R-ISS stage III predicted inferior response (HR 0.35, *p* = 0.04), whereas ≥3 prior lines of therapy correlated with shorter PFS (HR 1.77, *p* = 0.012). Adverse events were predominantly hematologic, with limited grade 3–4 toxicity and no treatment-related mortality. **Conclusions**: This multicenter real-world analysis clarifies the relative positioning of commonly used pomalidomide-based regimens in RRMM and underscores the importance of treatment timing and disease stage in optimizing outcomes.

## Simple Summary

Selecting optimal pomalidomide-based therapy for relapsed or refractory multiple myeloma (RRMM) remains challenging due to limited comparative data. In this multicenter real-world study of 230 patients, we compared outcomes among pomalidomide combined with bortezomib or ixazomib (V/IPD), carfilzomib (KPD), or daratumumab (DPD). Daratumumab-based therapy was associated with higher response rates and deeper remissions, although progression-free survival was comparable across regimens. Advanced disease stage and multiple prior lines of therapy were linked to inferior outcomes. These findings provide practical evidence to support regimen selection in routine clinical practice and highlight the importance of disease stage and treatment timing when choosing pomalidomide-based combinations for RRMM.

## 1. Introduction

Multiple myeloma (MM) is a clonal plasma cell disorder characterized by the uncontrolled proliferation of malignant plasma cells in the bone marrow and associated organ damage [1]. Over the past two decades, advanced therapies, including proteasome inhibitors (PIs), immunomodulatory drugs (IMiDs), and antibody-based or cellular immunotherapies, have significantly improved patient survival [2]. Despite these advances, nearly all patients eventually experience relapse or develop refractory disease. IMiDs serve as a cornerstone of MM therapy, acting through cereblon-mediated degradation of Ikaros and Aiolos, which promotes apoptosis of myeloma cells and enhances antitumor immune responses [3]. Pomalidomide, a third-generation IMiD, has demonstrated potent antimyeloma activity and favorable tolerability, making it an effective treatment option for relapsed or refractory multiple myeloma (RRMM), particularly among patients refractory to lenalidomide [4,5].

Pomalidomide received approval from the U.S. Food and Drug Administration (FDA) in 2013 and from the China National Medical Products Administration (NMPA) in 2020 for the treatment of RRMM. Pomalidomide has been widely used in combination with PIs (Bortezomib, Carfizomib or Ixazomibe) or anti-CD38 monoclonal antibodies (Daratumumab or Isatuximab), showing substantial clinical benefit in Bortezomib or Lenalidomide-refractory disease. In a phase II study of Carfilzomib/Pomalidomide/Dexamethasone (KPD) as second-line therapy after lenalidomide maintenance failure, 75% of patients achieved very good partial response (VGPR) or better, with median progression free survival (PFS) of 26 months in transplanted and 17 months in non-transplanted patients [6]. The MM-014 study confirmed durable responses with Daratumumab/Pomalidomide/Dexamethasone (DPD) in lenalidomide-exposed patients (ORR 77.7%, median PFS 30.8 months), while the APOLLO trial demonstrated a significant PFS improvement with the addition of daratumumab to pomalidomide/dexamethasone [7,8]. Based on these clinical trial data, pomalidomide-based regimens have been incorporated as preferred options in the latest NCCN Guidelines for patients with RRMM after one to three prior lines of therapy [9].

However, real-world clinical practice often differs from controlled trial settings, as treatment decisions are influenced by multiple patient- and disease-related factors such as age, cytogenetic abnormalities, comorbidities, and access to therapies. Therefore, real-world studies are essential to evaluate the effectiveness and tolerability of pomalidomide-based regimens in routine clinical settings and to inform evidence-based treatment decisions. Here, we conducted a multicenter retrospective study to evaluate the clinical outcomes of pomalidomide-based regimens in patients with relapsed or refractory multiple myeloma (RRMM) across 12 institutions in China. This study evaluated treatment patterns, efficacy, and safety in real-world practice and compared different pomalidomide-based combinations in terms of therapeutic response and progression-free survival.

## 2. Materials and Methods

### 2.1. Study Design and Patients

This was a multicenter, retrospective study conducted across 12 medical centers in China. A total of 230 patients with RRMM who received pomalidomide-based regimens between October 2019 and April 2025 were included in the analysis. The inclusion criteria were as follows: patients aged 18 years or older who were diagnosed with multiple myeloma according to the International Myeloma Working Group (IMWG) criteria [10] had received at least one prior line of therapy, were treated with at least one cycle of a pomalidomide-based regimen, and had sufficient clinical data available for analysis, including baseline characteristics, treatment information, response assessment, and follow-up data. Patients diagnosed with smoldering multiple myeloma, plasma cell leukemia, or solitary plasmacytoma were excluded. Clinical data, treatment response, cytogenetic profile, survival outcomes and adverse events (AEs) were retrospectively collected from electronic medical records at each participating center. Information regarding prior exposure to monoclonal antibodies, including daratumumab, was not uniformly available across participating centers and was therefore not included in the analysis. The study protocol was approved by the institutional medical ethics committee, and written informed consent was obtained from all patients prior to treatment (Ethical approval No. K2024020-00).

### 2.2. Pomalidomide-Based Regimens

Patients received Pomalidomide in combination with Bortezomib (VPD), Ixazomib (IPD), Carfilzomib (KPD), or Daratumumab (DPD), together with dexamethasone, according to institutional practice and physician’s discretion. As Bortezomib and Ixazomib are both boronate-based and reversible proteasome inhibitors with similar β5 subunit selectivity [11], VPD and IPD regimens were combined and analyzed together as the V/IPD group. Pomalidomide was administered orally at a daily dose of 2–4 mg on days 1–21 of each 28-day cycle, with the starting dose determined by patient age, prior treatment history, and tolerance. Dexamethasone was given weekly at a dose of 20–40 mg, adjusted as needed. The dosing and schedule of proteasome inhibitors or monoclonal antibodies followed standard clinical protocols [9]. Dose reductions, temporary interruptions, or discontinuation were permitted in cases of hematologic or non-hematologic toxicities based on physician judgment. Patients who received more than one pomalidomide-based regimen were classified according to the first pomalidomide-containing regimen administered. Supportive care and prophylactic measures, including bone protection, renal protection, and management of adverse events, were provided according to institutional practice and physician discretion.

### 2.3. Efficacy and Safety Outcomes

The primary endpoint of this study was the objective response rate (ORR), and the secondary endpoints included progression-free survival (PFS) and safety. Treatment response was evaluated according to the IMWG criteria [12], including complete response (CR), very good partial response (VGPR), partial response (PR), stable disease (SD), and progressive disease (PD). In this retrospective real-world cohort, response assessments were based on available clinical records. Although CR was defined according to IMWG criteria, bone marrow assessment was not uniformly performed in all patients in routine practice. ORR was defined as the proportion of patients achieving CR, VGPR, or PR. Efficacy was assessed every one to two treatment cycles or whenever disease relapse was suspected, and the best overall response achieved during pomalidomide-based therapy was recorded as the treatment outcome. PFS was calculated from the initiation of pomalidomide-based therapy to disease progression or death from any cause, whichever occurred first, and patients who were alive or progression-free at the last follow-up (18 August 2025) were censored. Adverse events (AEs) were evaluated and graded according to the National Cancer Institute Common Terminology Criteria for Adverse Events (CTCAE, version 5.0) [13]. High-risk cytogenetics was defined, based on IMWG recommendations [14], as the presence of del(17p), t(4;14), t(14;16), and t(14;20), or the gain of 1q detected by fluorescence in situ hybridization (FISH). TP53 mutation status was not routinely available and therefore not included in the high-risk definition.

### 2.4. Statistical Analysis

Descriptive statistics were used to summarize baseline patient characteristics and clinical outcomes, with categorical variables presented as numbers and percentages (*n*, %) and continuous variables as medians and ranges. Group differences in ORR and the distribution of response categories (CR, VGPR, PR, SD, and PD) among the three treatment groups were compared using the chi-square test, with Bonferroni correction applied for multiple pairwise comparisons. PFS was estimated using the Kaplan–Meier method, and differences among treatment groups were evaluated using the log-rank test with Bonferroni adjustment. Univariate and multivariate analyses were conducted to identify factors associated with ORR and PFS, with variables showing *p* < 0.1 in univariate analysis included in multivariate models. Logistic regression was used for ORR, and Cox proportional hazards regression was applied for PFS. All *p* values were two-sided, and *p* < 0.05 was considered statistically significant. Statistical analyses were performed using SPSS Statistics ver. 26.0 (IBM Corp., Armonk, NY, USA) and GraphPad Prism ver. 9.0 (GraphPad Software, San Diego, CA, USA).

## 3. Results

### 3.1. Patient Baseline Characteristics and Treatment History

Based on the treatment regimen, patients were categorized into three groups: V/IPD (*n* = 66), KPD (*n* = 69), and DPD (*n* = 95). Baseline disease characteristics and prior treatment history are summarized in Table 1, and their distribution according to treatment regimen is presented in Table S1.

The median age at diagnosis was 62 years (range, 38–83), and 57.4% of patients were male. Nearly half of the patients (49.1%) were classified as ISS stage III, and 32.2% as R-ISS stage III. High-risk cytogenetic abnormalities were identified in 47.4% of patients. Pomalidomide-based therapy was initiated a median of 2.7 years (range, 0.2–22.3) after diagnosis, and 42.2% of patients received the regimen at first relapse. Almost all patients had been exposed to both proteasome inhibitors (99.1%) and immunomodulatory agents (90.0%) in prior treatment lines. The proportions of patients refractory to Bortezomib, Ixazomib, and Lenalidomide were 64.3%, 25.7%, and 79.6%, respectively. A total of 23.9% of patients had previously undergone autologous stem-cell transplantation (ASCT).

The three treatment groups were generally comparable in age, sex, ISS/R-ISS stage, and cytogenetic risk category. Patients receiving KPD or DPD regimens had received treatment in later lines compared with those treated with V/IPD (*p* = 0.033). Regarding prior therapy exposure, patients in the DPD group had lower rates of previous Bortezomib (*p* = 0.035) use but higher rates of Ixazomib (*p* = 0.001) exposure compared with the other groups. The proportions of patients refractory to Bortezomib (*p* < 0.001), Ixazomib (*p* < 0.001), and Lenalidomide (*p* = 0.007) were lower in the V/IPD group than in the KPD and DPD groups. The prevalence of high-risk cytogenetic abnormalities and the proportion of patients who had undergone prior ASCT were similar among the three groups.

### 3.2. Response to Pomalidomide-Based Therapy

The median duration of pomalidomide-based therapy was 10.8 months (range, 1.0–43.6), with a median of 12 cycles (range, 1–47) administered. Treatment duration was calculated from the initiation of the regimen to treatment discontinuation, regardless of subsequent transition to Pomalidomide based maintenance therapy. Treatment was discontinued because of disease progression in 130 patients (V/IPD 43, KPD 35, DPD 52), due to adverse events in 6 (V/IPD 1, KPD 1, DPD 4), and by patient withdrawal in 3 (V/IPD 2, KPD 0, DPD 1). Response data were available for 221 of the 230 enrolled patients, with nine patients (V/IPD = 4, KPD = 3, DPD = 2) excluded from response analysis because of unavailable response records.

**Figure 1:**
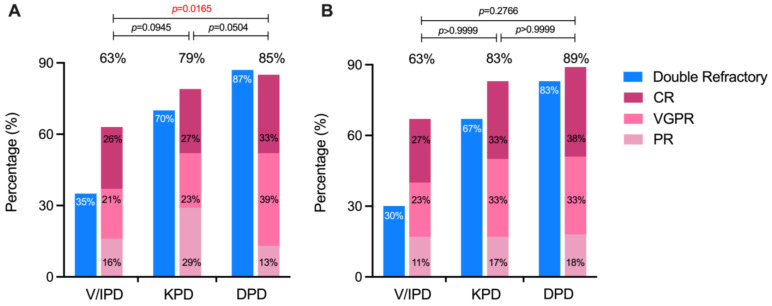
Overall response rates (ORR) according to pomalidomide-based regimens in the overall cohort (**A**) and in the first-relapse subgroup (**B**). Bars represent the proportions of patients achieving partial response (PR), very good partial response (VGPR), and complete response (CR). Percentages above each bar indicate ORRs. Pairwise comparisons between regimens are shown above the panels. V/IPD, pomalidomide plus bortezomib or ixazomib; KPD, pomalidomide plus carfilzomib; DPD, pomalidomide plus daratumumab.

As shown in Table 2, the ORR for the entire cohort was 73.9%, including CR in 28.3%, VGPR in 27.8%, and PR in 17.8%. Among patients who received pomalidomide-based therapy at first relapse, the ORR was 77.3% (CR 31.9%, VGPR 28.9%, PR 16.5%). The distribution of response categories differed significantly between the DPD and V/IPD groups (*p* = 0.0165), driven by a higher proportion of deeper responses in the DPD group (CR + VGPR, 72% vs. 47%), whereas no significant differences were observed among the other pairwise comparisons (Figure 1A). A similar trend was observed among patients who received Pomalidomide-based therapy at first relapse (Figure 1B), although the difference did not reach statistical significance. Despite a higher proportion of double-refractory cases in the DPD and KPD groups compared with the V/IPD group, both regimens achieved high ORRs.

To further explore factors influencing treatment response, univariate and multivariate logistic regression analyses were performed and shown in Table 3. Univariate analysis showed that KPD and DPD regimens were associated with higher ORRs compared with V/IPD, while the IgG subtype, R-ISS stage III, and HRCA were related to lower ORRs. After multivariate analysis, the DPD regimen and R-ISS stage III emerged as independent predictors of response, with DPD associated with a significantly higher likelihood of achieving the ORR (OR = 4.83, 95% CI 2.05–11.36, *p* < 0.001), whereas R-ISS stage III associated with a reduced probability of the ORR (OR = 0.35, 95% CI: 0.17–0.71, *p* = 0.004).

### 3.3. Progression-Free Survival

**Figure 2:**
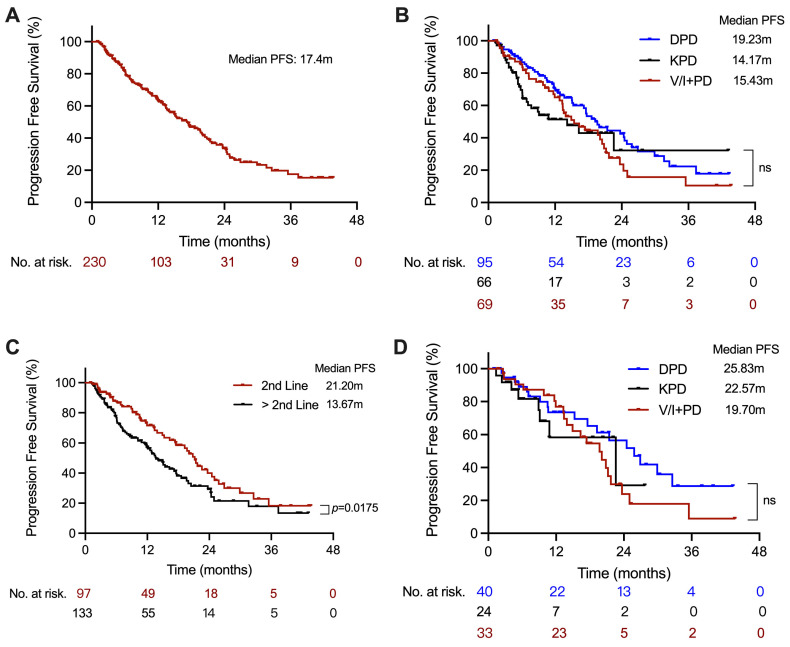
Kaplan–Meier curves for progression-free survival (PFS) in the overall cohort (**A**), the overall cohort stratified by regimen (**B**), patients receiving second-line versus later-line therapy (**C**), and the first-relapse subgroup stratified by regimen (**D**). Log-rank tests were used to evaluate differences between groups. V/IPD, pomalidomide plus bortezomib or ixazomib; KPD, pomalidomide plus carfilzomib; DPD, pomalidomide plus daratumumab; ns, not significant.

The median follow-up duration was 19.4 months (95% CI: 16.5–22.3) from Pomalidomide-based treatment initiation to the last follow-up. Overall, patients experienced a median PFS of 17.4 months (95% CI: 13.7–20.1) (Figure 2A). When stratified by treatment regimen, PFS was 19.2 months (95% CI: 15.1–24.9) in the DPD group, 14.2 months (95% CI: 6.9–not estimable) in the KPD group, and 15.4 months (95% CI: 12.8–20.5) in the V/IPD group, with no statistically significant difference among the three groups (Figure 2B). Patients who received pomalidomide-based therapy at first relapse achieved significantly longer PFS than those treated in later lines (21.2 vs. 13.7 months, *p* = 0.0175; Figure 2C). Within the first-relapse subgroup, PFS was 25.8 months (95% CI: 17.6–32.6) in the DPD group, 22.6 months (95% CI: 8.9–not estimable) in the KPD group, and 19.7 months (95% CI: 13.4–21.7) in the V/IPD group, with no significant intergroup difference (Figure 2D). Specially, Pairwise log-rank comparisons showed no significant difference between KPD and DPD (*p* = 0.064).

To identify factors independently associated with PFS, univariate and multivariate Cox regression analyses were performed (Table 4). In the univariate analysis, patients who had received ≥3 prior lines of therapy showed significantly shorter PFS (HR = 1.72, 95% CI 1.18–2.51, *p* = 0.005). R-ISS stage III was also entered into the multivariate model for adjustment. In the multivariate analysis, ≥3 prior lines of therapy remained an independent predictor of poorer PFS (HR = 1.77, 95% CI 1.13–2.77, *p* = 0.012), whereas treatment regimen, cytogenetic risk, and R-ISS stage were not significantly associated with PFS.

### 3.4. Safety and Tolerability

Treatment-related adverse events (AEs) are summarized in Table 5. The hematologic AEs included anemia (20.0%), neutropenia (19.1%), and thrombocytopenia (14.8%), with grade 3–4 events observed in 4.3%, 7.8%, and 4.3% of patients, respectively. The most frequent non-hematologic toxicities were pneumonia (16.1%) and nausea (4.3%). Grade 3 pneumonia occurred in 7.8% of patients, and one case (0.4%) of grade 4 infection was recorded. Other non-hematologic AEs, including venous thrombosis, rash, and gastrointestinal symptoms, were infrequent (<5%) and generally mild (grade 1–2). No treatment-related deaths were observed.

## 4. Discussion

This multicenter retrospective study reflects routine clinical practice in patients with RRMM treated with pomalidomide-based regimens after prior exposure to proteasome inhibitors and/or immunomodulatory agents. In a cohort characterized by a high prevalence of double-refractory disease and nearly half with high-risk cytogenetics, pomalidomide-containing combinations achieved an ORR of 73.9% and a median PFS of 17.4 months, with favorable tolerability. Furthermore, multivariate analyses demonstrated differential response probabilities among regimen categories and R-ISS stage. Treatment line remained an independent determinant of PFS. Together, these findings provide practical insights into regimen selection and therapeutic sequencing in routine practice for RRMM.

Pomalidomide combined with proteasome inhibitors has demonstrated synergistic antimyeloma activity [15,16,17] and has been widely incorporated into treatment strategies for relapsed disease. In the final analysis of the phase III OPTIMISMM trial, VPD achieved an ORR of 82.2% with a median PFS of 22.1 months in lenalidomide-exposed patients [18]. The earlier analysis reported that patients treated at first relapse achieved an ORR of 90.1% with a median PFS of 20.7 months [19]. Similarly, in the Alliance A061202 study evaluating IPD in RRMM patients, the overall ORR was 51.7% with a median PFS of 16.8 months [20], while patients treated at first relapse achieved an ORR of 63.2% and a median PFS of 20.3 months [21]. However, real-world data specifically evaluating pomalidomide in combination with bortezomib or ixazomib remain limited. In our cohort, the V/IPD subgroup achieved an ORR of 63% and a median PFS of 15.4 months. Among patients treated at first relapse, the median PFS improved to 19.7 months, whereas the ORR remained similar. Our response rate was lower than that reported in OPTIMISMM, while closely aligning with the efficacy observed in Alliance A061202. This pattern is plausibly related to case mix in our cohort, which included double-refractory disease, more advanced treatment lines, and less stringently selected patients with inferior functional status, and thus offers an effectiveness estimate for V/IPD outside trial eligibility constraints. In addition, from a real-world perspective, PI-based combinations such as V/IPD may represent more accessible treatment options in certain settings, particularly when treatment affordability is a relevant consideration, while still providing clinically meaningful efficacy.

Carfilzomib, a second-generation proteasome inhibitor, has been widely incorporated into treatment strategies for relapsed/refractory multiple myeloma, including in combination with pomalidomide and dexamethasone [9]. In the phase 2 SELECT trial [22], KPD achieved an ORR of 58% and a median PFS of 11.1 months in patients treated at first or second relapse. Two real-world studies from China reported ORRs of 86.8% (*n* = 38) [23] and 66.5% (*n* = 81) [24], with corresponding median PFS of 13.4 months and 12.7 months, respectively. These results illustrate the heterogeneity of reported outcomes across studies and underscore the influence of treatment line and patient selection on efficacy estimates. In our cohort, KPD resulted in an ORR of 79% and a median PFS of 14.2 months overall. Outcomes were more favorable in the first-relapse subgroup, with an ORR of 83% and a median PFS of 22.6 months. Similarly, the EMN011/HOVON114 trial [25] demonstrated high efficacy in the first-relapse setting, with an ORR of 92% and a median PFS of 27 months, supporting earlier use of KPD in appropriate patients. These data collectively underscore the line-dependent efficacy of KPD, with more pronounced benefit observed when administered at first relapse.

The other pomalidomide-based regimen, DPD, has demonstrated consistent activity in RRMM across both single-arm and randomized studies. In MM-014, DPD achieved an ORR of 78.6% with a median PFS of 23.7 months [5], while APOLLO reported an ORR of 69% for the daratumumab-containing arm [8]. In our cohort, DPD resulted in an ORR of 85% and a median PFS of 19.2 months overall, with more favorable outcomes at first relapse (ORR 89%, median PFS 25.8 months). A separate real-world study from China (*n* = 24) similarly reported an ORR of 75% but a median PFS of 6.7 months [26]. Notably, both real-world cohorts demonstrated response rates not inferior to those reported in clinical trials, whereas progression-free survival was comparatively shorter. Although the ORR of DPD in our real-world cohort was not inferior to that reported in clinical trials, PFS was comparatively shorter. This discrepancy may be attributable to differences in treatment adherence and adverse event management outside controlled trial settings, which can limit sustained therapy exposure. Consistent with the preceding observations, multivariable analyses further confirmed that DPD was independently associated with improved ORR, whereas no independent advantage was observed with respect to PFS. Given the lack of direct head-to-head comparisons among different pomalidomide-based regimens, additional evidence is warranted to further validate these comparative observations. Accordingly, therapeutic decisions regarding DPD in clinical practice require integration of treatment cost, patient fitness, and infection risk in addition to depth of response, particularly given the potentially higher economic burden associated with daratumumab-based regimens.

Beyond regimen selection, established clinical and biological risk factors continued to influence outcomes. With respect to progression-free survival, receipt of ≥3 prior lines of therapy was independently associated with inferior outcomes (HR 1.77, *p* = 0.012). This finding aligns with previous real-world studies reporting declining response rates and progressively shorter PFS with increasing lines of therapy [27,28], underscoring the clinical importance of earlier therapeutic intervention. The persistence of R-ISS stage III as an independent predictor of inferior response indicates that, within pomalidomide-based regimens, R-ISS rather than high-risk cytogenetic abnormalities retains independent prognostic value in RRMM. High-risk cytogenetic abnormalities were associated with response in univariable analysis but did not retain independent significance after multivariable adjustment. Previous data on the prognostic impact of high-risk cytogenetic abnormalities in pomalidomide-based regimens remain heterogeneous, with some studies demonstrating adverse associations [26,29] while others fail to confirm independent significance [30,31]. In this context, the clinical prognostic value of high-risk cytogenetics cannot be conclusively established and warrants further evaluation in adequately powered cohorts.

Several limitations should be acknowledged. First, the retrospective design and multicenter nature of this study may introduce heterogeneity in treatment practices and data collection. In particular, treatment allocation was not randomized and was influenced by clinical factors such as prior therapy exposure, treatment line, and patient condition, which may introduce potential selection bias and confounding. Although we attempted to account for these factors through multivariable analyses and subgroup analyses, residual confounding cannot be fully excluded. In addition, Similarly, overall survival (OS) data were immature due to limited follow-up, and duration of response (DoR) could not be reliably assessed because of incomplete and heterogeneous documentation of response timing across centers. Therefore, these outcomes should be interpreted with caution. Improved standardization of adverse event reporting and clinical data collection in clinical practice may enable more precise assessment of treatment efficacy and treatment tolerability, particularly for endpoints such as DoR and lower-grade (grade 1–2) toxicities.

Overall, this multicenter real-world analysis clarifies the clinical positioning of commonly used pomalidomide-based combinations in RRMM. V/IPD demonstrated moderate response rates and durability and may remain a reasonable option in selected patients refractory to lenalidomide but not to proteasome inhibitors. In this context, both KPD and DPD were active in patients refractory to both bortezomib and lenalidomide. While DPD achieved higher ORR than KPD, PFS was comparable between the two regimens. These data support the rational use of pomalidomide-based combinations across different relapse settings, with particular consideration of prior treatment exposure and disease stage.

## 5. Conclusions

In this multicenter real-world study, commonly used pomalidomide-based combinations demonstrated meaningful clinical activity in RRMM. V/IPD showed moderate response and durability, whereas both KPD and DPD were active in patients refractory to bortezomib and lenalidomide. Although DPD achieved higher response rates, progression-free survival was comparable between regimens. These findings support the rational selection of pomalidomide-based combinations according to prior treatment exposure and disease stage.

## Figures and Tables

**Table 1:** Baseline disease characteristics and prior treatment history.

	Total (*n* = 230)
**Age, median (range), y**	62 (38–83)
<65	132 (57.4)
65 to <75	77 (33.5)
≥75	21 (9.1)
**Sex**	
Male	132 (57.4)
Female	98 (42.6)
**Type of Myeloma**	
IgG	107 (46.5)
IgA	45 (19.6)
IgD	16 (6.9)
Light chain	59 (25.7)
Non-secretory	3 (3.0)
**ISS Stage**	
I	42 (18.3)
II	68 (29.6)
III	113 (49.1)
NA	7 (3.0)
**R-ISS Stage**	
I	35 (15.2)
II	109 (47.4)
III	74 (32.2)
NA	12 (5.2)
**Cytogenetic Abnormalities**	
High Risk	109 (47.4)
Standard Risk or NA	121 (52.6)
Year since initial diagnosis, median (range)	2.7 (0.2–22.3)
**Prior lines of therapy**	
1	97 (42.2)
2	65 (28.3)
3	35 (15.2)
>3	33 (14.3)
**Prior therapy**	
PIs + IMiDs	207 (90.0)
PIs	228 (99.1)
Bortezomib	221 (96.1)
Ixazomib	80 (34.8)
IMiDs	207 (90.0)
Lenalidomide	206 (89.6)
Thalidomide	4 (1.7)
**Refractory to**	
Bortezomib	148 (64.3)
Ixazomib	59 (25.7)
Lenalidomide	183 (79.6)
**Prior ASCT**	55 (23.9)

Values represent *n* (%) of patients unless otherwise indicated. Total percentages within categories may not equal 100% due to rounding. ASCT, autologous stem cell transplantation.

**Table 2:** Treatment Response to Pomalidomide-Based Regimen.

	Total (*n* = 230)	2nd Line (*n* = 97)
Overall response *	170 (73.9)	75 (77.3)
Complete response	65 (28.3)	31 (31.9)
Very good partial response	64 (27.8)	28 (28.9)
Partial response	41 (17.8)	16 (16.5)
Stable disease	33 (14.4)	12 (12.4)
Progressive disease	18 (7.8)	7 (7.2)
Not assessable	9 (3.9)	3 (3.1)

Data are presented as *n* (%). * Overall response was defined as patients who achieved a best response of PR or better.

**Table 3:** Univariate and Multivariate logistic regression for ORRs.

Variables	Univariate OR (95% CI)	*p*-Value	Multivariate OR (95% CI)	*p*-Value
Regimen				
KPD vs. VPD/IPD	2.19 (1.01–4.80)	0.049	2.28 (0.99–5.22)	0.051
DPD vs. VPD/IPD	3.33 (1.55–7.17)	0.002	4.83 (2.05–11.36)	<0.001
Age ≥ 65	0.87 (0.47–1.64)	0.673		
Sex (Female)	1.52 (0.81–2.85)	0.190		
Type of MM (IgG)	0.47 (0.25–0.898)	0.022	0.50 (0.25–1.01)	0.053
ISS stage III	0.61 (0.32–1.17)	0.138		
R-ISS stage III	0.41 (0.21–0.79)	0.008	0.35 (0.17–0.71)	0.004
HRCA	0.44 (0.19–1.02)	0.055	1.22 (0.44–3.35)	0.699
≥2 HRCA	0.73 (0.29–1.87)	0.520		
Lenalidomide refractory	1.43 (0.65–3.12)	0.375		
PI refractory	1.64 (0.82–3.29)	0.161		
Double refractory	1.35 (0.70–2.59)	0.372		
ASCT	1.39 (0.64–3.01)	0.406		
≥3 prior lines of therapy	0.72 (0.37–1.40)	0.335		

OR, odds ratio; PI, proteasome inhibitor; HRCA, high risk cytogenetic abnormalities; ASCT, autogenetic stem cell transplantation. Covariates demonstrating a *p*-value < 0.10 in univariate Logistic regression were selected for inclusion in the multivariate model to identify independent prognostic factors. OR > 1 indicates an increased likelihood of achieving overall response, whereas OR < 1 indicates a decreased likelihood.

**Table 4:** Univariate and Multivariate Cox regression for PFS.

Variables	Univariate HR (95% CI)	*p*-Value	Multivariate HR (95% CI)	*p*-Value
Regimen				
KPD vs. VPD/IPD	1.16 (0.72–1.86)	0.545		
DPD vs. VPD/IPD	0.72 (0.47–1.09)	0.121		
KPD vs. DPD				
Age ≥ 65	1.03 (0.72–1.47)	0.873		
Sex (Female)	0.96 (0.66–1.38)	0.822		
Type of MM (IgG)	0.94 (0.82–1.07)	0.321		
ISS stage III	1.11 (0.78–1.60)	0.562		
R-ISS stage III	1.44 (0.98–2.11)	0.061	1.25 (0.81–1.92)	0.312
HRCA	1.34 (0.89–2.02)	0.162		
≥2 HRCA	1.16 (0.70–1.91)	0.573		
Lenalidomide refractory	0.99 (0.60–1.61)	0.959		
PI refractory	1.09 (0.70–1.68)	0.705		
Double refractory	0.95 (0.65–1.40)	0.812		
ASCT	0.90 (0.60–1.35)	0.608		
≥3 prior lines of therapy	1.72 (1.18–2.51)	0.005	1.77 (1.13–2.77)	0.012

PI, proteasome inhibitor; ASCT, autogenetic stem cell transplantation. Covariates demonstrating a *p*-value < 0.20 in univariate Cox regression were selected for inclusion in the multivariate model to identify independent prognostic factors. HR > 1 indicates an increased risk of progression or death (worse PFS), whereas HR < 1 indicates a reduced risk (better PFS).

**Table 5:** Hematological and non-hematological toxicities.

Toxicity	Episodes, *n* (%) Total	Grade 3	Grade 4
Neutropenia	44 (19.1)	17 (7.4)	1 (0.4)
Thrombocytopenia	34 (14.8)	10 (4.3)	0
Anemia	46 (20.0)	10 (4.3)	0
Pneumonia	37 (16.1)	18 (7.8)	0
Other infection	6 (2.6)	0	1 (0.4)
Venous thrombosis	3 (1.3)	0	0
ALT increase	3 (1.3)	0	0
Fatigue	4 (1.7)	2 (0.8)	0
Nausea	10 (4.3)	0	0
Constipation	1 (0.4)	0	0
Diarrhea	3 (1.3)	0	0
Rash	8 (3.5)	0	0

## Data Availability

The data supporting this study’s findings are not publicly available due to containing information that could compromise the privacy of research participants; nevertheless, some data are available from corresponding author.

## Supplementary Materials

The following supporting information can be downloaded at: https://www.mdpi.com/article/10.3390/cancers18071160/s1, Table S1: Baseline disease characteristics and prior treatment history for 3 regimen groups.

## References

[1] Rajkumar S.V. (2018). Multiple myeloma: 2018 update on diagnosis, risk-stratification, and management. Am. J. Hematol..

[2] Cowan A.J., Green D.J., Kwok M., Lee S., Coffey D.G., Holmberg L.A., Tuazon S., Gopal A.K., Libby E.N. (2022). Diagnosis and Management of Multiple Myeloma: A Review. JAMA.

[3] Holstein S.A., McCarthy P.L. (2017). Immunomodulatory Drugs in Multiple Myeloma: Mechanisms of Action and Clinical Experience. Drugs.

[4] Davies F.E., Leleu X., Vogel P., Dhanasiri S., Le Nouveau P., Weisel K. (2023). A Meta-Analysis of the Efficacy of Pomalidomide-Based Regimens for the Treatment of Relapsed/Refractory Multiple Myeloma After Lenalidomide Exposure. Clin. Lymphoma Myeloma Leuk..

[5] Bahlis N.J., Samaras C., Reece D., Sebag M., Matous J., Berdeja J.G., Shustik J., Schiller G.J., Ganguly S., Song K. (2024). Pomalidomide/Daratumumab/Dexamethasone in Relapsed or Refractory Multiple Myeloma: Final Overall Survival from MM-014. Clin. Lymphoma Myeloma Leuk..

[6] Sonneveld P., Zweegman S., Cavo M., Nasserinejad K., Broijl A., Troia R., Pour L., Croockewit S., Corradini P., Patriarca F. (2022). Carfilzomib, Pomalidomide, and Dexamethasone As Second-line Therapy for Lenalidomide-refractory Multiple Myeloma. Hemasphere.

[7] Bahlis N.J., Siegel D.S., Schiller G.J., Samaras C., Sebag M., Berdeja J., Ganguly S., Matous J., Song K., Seet C.S. (2022). Pomalidomide, dexamethasone, and daratumumab immediately after lenalidomide-based treatment in patients with multiple myeloma: Updated efficacy, safety, and health-related quality of life results from the phase 2 MM-014 trial. Leuk. Lymphoma.

[8] Dimopoulos M.A., Terpos E., Boccadoro M., Delimpasi S., Beksac M., Katodritou E., Moreau P., Baldini L., Symeonidis A., Bila J. (2023). Subcutaneous daratumumab plus pomalidomide and dexamethasone versus pomalidomide and dexamethasone in patients with relapsed or refractory multiple myeloma (APOLLO): Extended follow up of an open-label, randomised, multicentre, phase 3 trial. Lancet Haematol..

[9] Network NCC (2025). NCCN Clinical Practice Guidelines in Oncology: Multiple Myeloma.

[10] Rajkumar S.V., Dimopoulos M.A., Palumbo A., Blade J., Merlini G., Mateos M.-V., Kumar S., Hillengass J., Kastritis E., Richardson P. (2014). International Myeloma Working Group updated criteria for the diagnosis of multiple myeloma. Lancet Oncol..

[11] Ito S. (2020). Proteasome Inhibitors for the Treatment of Multiple Myeloma. Cancers.

[12] Durie B.G., Harousseau J.L., Miguel J.S., Blade J., Barlogie B., Anderson K., Gertz M., Dimopoulos M., Westin J., Sonneveld P. (2006). International uniform response criteria for multiple myeloma. Leukemia.

[13] U.S. Department of Health and Human Services NioH, National Cancer Institute (2017). Common Terminology Criteria for Adverse Events (CTCAE).

[14] Sonneveld P., Avet-Loiseau H., Lonial S., Usmani S., Siegel D., Anderson K.C., Chng W.-J., Moreau P., Attal M., Kyle R.A. (2016). Treatment of multiple myeloma with high-risk cytogenetics: A consensus of the International Myeloma Working Group. Blood.

[15] Borsi E., Martello M., Santacroce B., Zamagni E., Tacchetti P., Pantani L., Mancuso K., Rocchi S., Cavo M., Terragna C. (2018). Treatment optimization for multiple myeloma: Schedule-dependent synergistic cytotoxicity of pomalidomide and carfilzomib in in vitro and ex vivo models. Haematologica.

[16] Das D.S., Ray A., Song Y., Richardson P., Trikha M., Chauhan D., Anderson K.C. (2015). Synergistic anti-myeloma activity of the proteasome inhibitor marizomib and the IM iD^®^ immunomodulatory drug pomalidomide. Br. J. Haematol..

[17] Katz M., Bjorklund C.C., Thakurta A., Serbina N. (2018). Mechanistic insights of pomalidomide activity in combination with bortezomib and dexamethasone in multiple myeloma and immune cells. Blood.

[18] Richardson P., Beksaç M., Oriol A., Lindsay J., Schjesvold F., Galli M., Yağcı M., Larocca A., Weisel K., Yu X. (2025). Pomalidomide, Bortezomib, and Dexamethasone Versus Bortezomib and Dexamethasone in Relapsed or Refractory Multiple Myeloma: Final Survival and Subgroup Analyses From the OPTIMISMM Trial. Eur. J. Haematol..

[19] Dimopoulos M., Weisel K., Moreau P., Anderson L.D., White D., San-Miguel J., Sonneveld P., Engelhardt M., Jenner M., Corso A. (2021). Pomalidomide, bortezomib, and dexamethasone for multiple myeloma previously treated with lenalidomide (OPTIMISMM): Outcomes by prior treatment at first relapse. Leukemia.

[20] Voorhees P.M., Suman V.J., Tuchman S.A., Laubach J.P., Hassoun H., Efebera Y.A., Mulkey F., Bova-Solem M., Santo K., Carlisle D. (2021). A phase I/II study of ixazomib, pomalidomide, and dexamethasone for lenalidomide and proteasome inhibitor refractory multiple myeloma (Alliance A061202). Am. J. Hematol..

[21] Voorhees P., Suman V., Efebera Y., Raje N., Tuchman S., Rodriguez C., Laubach J., Bova-Solem M., Carlisle D., Usmani S. (2024). Alliance A061202: Ixazomib, pomalidomide, and dexamethasone for patients with lenalidomide-refractory MM in first relapse. Blood Adv..

[22] Perrot A., Delimpasi S., Spanoudakis E., Frølund U., Belotti A., Oriol A., Moreau P., McFadden I., Xia Q., Arora M. (2024). An open-label phase 2 study treating patients with first or second relapse of multiple myeloma with carfilzomib, pomalidomide, and dexamethasone (KPd): SELECT study. Leuk. Lymphoma.

[23] Xiao X., Shao Y., Li X., Jiang H., Jiang W., Chen P., Xie J., Qian W. (2025). Carfilzomib plus pomalidomide and dexamethasone as salvage therapy in patients with relapsed or refractory multiple myeloma in China: A retrospective study. Transl. Cancer Res..

[24] Fan H., Wei R., Feng X., Zhang X., Li T., Liu L., Wang H., Sun L., Shi X., Zhao H. (2024). Real-World Effectiveness and Safety Analysis of Carfilzomib-Pomalidomide-Dexamethasone in Relapsed/Refractory Multiple Myeloma: A Multicenter Retrospective Study from Eastern Shandong of China. Blood.

[25] Sonneveld P., Zweegman S., Cavo M., Nasserinejad K., Broyl A., Troia R., Pour L., Croockewit S., Corradini P., Bos G.M. (2021). Carfilzomib, pomalidomide and dexamethasone (KPd) in patients with first progression of multiple myeloma refractory to bortezomib and lenalidomide. Final report of the EMN011/HOVON114 trial. Blood.

[26] Han X., Jiang X., He J., Zheng G., Xiong Y., Wen Y., Yang Y., He D., Chen Q., Zhao Y. (2024). Clinical outcomes of pomalidomide-based and daratumumab-based therapies in patients with relapsed/refractory multiple myeloma: A real-world cohort study in China. Cancer Med..

[27] LeBlanc R., Mian H., Reece D., Masih-Khan E., Kardjadj M., Jimenez-Zepeda V.H., McCurdy A., Song K., Sebag M., Louzada M. (2022). Outcomes of daratumumab in the treatment of multiple myeloma: A retrospective cohort study from the Canadian Myeloma Research Group Database. Br. J. Haematol..

[28] Atrash S., Thompson-Leduc P., Tai M.-H., Kaila S., Gray K., Ghelerter I., Lafeuille M.-H., Lefebvre P., Rossi A. (2021). Treatment patterns and effectiveness of patients with multiple myeloma initiating daratumumab across different lines of therapy: A real-world chart review study. BMC Cancer.

[29] Siegel D.S., Schiller G.J., Samaras C., Sebag M., Berdeja J., Ganguly S., Matous J., Song K., Seet C.S., Talamo G. (2020). Pomalidomide, dexamethasone, and daratumumab in relapsed refractory multiple myeloma after lenalidomide treatment. Leukemia.

[30] Maciocia N., Melville A., Cheesman S., Sharpley F., Ramasamy K., Streetly M., Jenner M., Benjamin R., Schey S., Maciocia P. (2017). Real-world use of pomalidomide and dexamethasone in double refractory multiple myeloma suggests benefit in renal impairment and adverse genetics: A multi-centre UK experience. Br. J. Haematol..

[31] Chari A., Suvannasankha A., Fay J.W., Arnulf B., Kaufman J.L., Ifthikharuddin J.J., Weiss B.M., Krishnan A., Lentzsch S., Comenzo R. (2017). Daratumumab plus pomalidomide and dexamethasone in relapsed and/or refractory multiple myeloma. Blood J. Am. Soc. Hematol..

