# Supplementary material for: Comparative Effectiveness of Pomalidomide-Based Regimens in Relapsed/Refractory Multiple Myeloma: A Multicenter Real-World Analysis in China
Source: Cancers (Basel). 2026 Apr 3;18(7):1160. doi: 10.3390/cancers18071160 (PMC13072356; doi:10.3390/cancers18071160)
Supplement: Supplementary file 1 [file cancers-18-01160-s001.zip › cancers-4198339-supplementary.pdf]

Table S1. Baseline disease characteristics and prior treatment history for 3 regimen groups.

	V/IPD (n =66)	KPD (n =69)	DPD (n =95)	p-Value
**Age, median (range), y**	63 (38-82)	62 (39-83)	64 (40-80)	0.828
<65	37 (56.1)	42 (60.9)	53 (55.8)	
65 to <75	23 (34.8)	22 (31.9)	32 (33.7)	
≥75	6 (9.1)	5 (7.2)	10 (10.5)	
**Sex, male/female**				0.398
Male	39 (59.1)	35 (50.7)	58 (61.1)	
Female	27 (40.9)	34 (49.3)	37 ()38.9	
**Type of Myeloma**				0.352
IgA	12 (18.2)	15 (21.7)	18 (18.9)	
IgG	29 (43.9)	36 (52.2)	42 (44.2)	
IgD	4 (6.1)	5 (7.2)	7 (7.4)	
Light chain	20 (30.3)	13 (18.8)	26 (27.4)	
Non-secretory	1 (1.5)	0	2 (2.1)	
**ISS Stage**				0.499
I	13 (19.7)	15 (21.7)	14 (14.7)	
II	20 (30.3)	20 (29.0)	28 (29.5)	
III	32 (48.5)	31 (44.9)	50 (52.6)	
NA	1 (1.5)	3 (4.4)	3 (3.2)	
**R-ISS Stage**				0.245
I	11 (16.7)	13 (18.8)	11 (11.6)	
II	33 (50.0)	30 (43.5)	46 (47.4)	
III	21 (31.8)	20 (29.0)	33 (34.7)	

NA	1 (1.5)	6 (8.7)	5 (5.3)	
**Cytogenetic Abnormalities**				0.763
High Risk	32 (48.5)	34 (49.3)	43 (45.2)	
Standard Risk or NA	33 (51.5)	35 (50.7)	52 (54.8)	
**Year since initial diagnostic, median (range)**	2.8 (0.2-22.2)	2.6 (0.2-21.2)	2.8 (0.2-22.3)	0.331
**Prior lines of therapy**				**0.033**
1	33(50.0)	24 (34.8)	40 (42.2)	
2	20(30.3)	18 (26.1)	27 (28.4)	
3	9(13.6)	10 (14.5)	16 (16.8)	
>3	4(6.1)	17 (24.6)	12 (12.6)	
**Prior therapy**				
PIs+ IMiDs	55 (83.3)	62 (89.9)	89 (93.7)	0.108
PIs				
Bortezomib	62 (93.9)	67 (97.1)	82 (86.3)	**0.035**
Ixazomib	19 (28.8)	15 (21.7)	46 (48.4)	**0.001**
IMiDs				
Lenalidomide	55 (83.8)	64 (92.8)	90 (94.7)	0.061
Thalidomide	1 (1.5)	2 (2.9)	1 (1.1)	0.330
**Refractory to**				
Bortezomib	18 (27.3)	55 (79.7)	75 (78.9)	**0.000**
Ixazomib	1 (1.5)	14 (20.3)	44 (46.3)	**0.000**
Lenalidomide	47 (71.2)	47 (68.1)	88 (92.6)	**0.007**
**Prior ASCT**	17 (25.8)	20 (29.0)	18 (18.9)	0.305

All the data presented as the number (percentage). ASCT, autogenetic stem cell transplantation. Bold values indicate statistically significant differences ($p < 0.05$).
